# Disseminated intravascular coagulation is strongly associated with severe acute kidney injury in patients with septic shock

**DOI:** 10.1186/s13613-023-01216-8

**Published:** 2023-12-01

**Authors:** Julie Helms, Hamid Merdji, Sébastien Loewert, François Severac, Alexandra Monnier, Julian Kaurin, Anaïs Curtiaud, Ferhat Meziani, Julien Demiselle

**Affiliations:** 1 Service de Médecine Intensive-Réanimation, Nouvel Hôpital Civil, Hôpitaux universitaires de Strasbourg, 1, Place de l’Hôpital, 67091 Strasbourg Cedex, France; 2 UMR 1260, Regenerative Nanomedicine (RNM), FMTS, INSERM (French National Institute of Health and Medical Research), Strasbourg, France; 3 Groupe Méthodes en Recherche Clinique (GMRC), Hôpital Civil, Hôpitaux universitaires de Strasbourg, Strasbourg, France

**Keywords:** Disseminated intravascular coagulation, Sepsis, Septic shock, Acute kidney injury, Acute kidney disease

## Abstract

**Background:**

Disseminated intravascular coagulation (DIC) worsens the prognosis of septic shock and contributes to multiple organ failure. To date, no data linking DIC and acute kidney injury (AKI) occurrence, severity, and evolution in this setting are available. We aimed at analyzing the association between AKI occurrence, severity and evolution in patients with septic shock-induced DIC. In a prospective monocentric cohort study, consecutive patients, 18 years and older, admitted in the ICU of Strasbourg University Hospital in the setting of systemic hypotension requiring vasopressor related to an infection, without history of terminal chronic kidney disease were eligible. AKI was defined according to the KDIGO classification. DIC diagnosis was based on the International Society on Thrombosis and Haemostasis (ISTH) score. Evolution of AKI was evaluated through the composite endpoint of major adverse kidney events. Only patients with DIC that occurred before or at the time of AKI diagnosis were considered. Univariate and multivariate analysis were performed to determine factors associated with renal outcomes.

**Results:**

350 patients were included, of whom 129 experienced DIC. Patients with DIC were more seriously ill (median SAPS II 64 vs. 56, *p* < 0.001), and had higher 28-day mortality (43.3% vs. 26.2%, *p* < 0.001). AKI was more frequent in patients with DIC (86.8% vs. 74.2%, *p* < 0.005), particularly for the more severe stage of AKI [KDIGO 3 in 58.1% of patients with DIC vs. 30.8% of patients without DIC, *p* < 0.001, AKI requiring renal replacement therapy (RRT) in 47.3% of patients with DIC vs. 21.3% of patients without DIC, *p* < 0.001]. After adjustment for confounding factors, DIC occurrence remained associated with the risk of having the more severe stage of AKI with an odds ratio (OR) of 2.74 [IC 95% (1.53–4.91), *p* < 0.001], and with the risk of requiring RRT during the ICU stay [OR 2.82 (1.53–5.2), *p* < 0.001].

**Conclusion:**

DIC appears to be strongly associated with the risk of developing the more severe form of AKI (stage 3 of the KDIGO classification, RRT requirement), even after adjustment for severity and other relevant factors.

**Supplementary Information:**

The online version contains supplementary material available at 10.1186/s13613-023-01216-8.

## Background

Acute kidney injury (AKI) in the intensive care unit (ICU) is associated with a higher mortality rate, with a close relationship between AKI severity and death [1, 2]. AKI has also been reported to be associated with worse long-term outcome: patients who experienced AKI during ICU stay are at higher risk of developing chronic kidney disease (CKD) and major cardiovascular events [3, 4].

Beyond common factors associated with AKI in ICU, septic shock remains the main trigger of AKI [5]. However, pathophysiology of sepsis-induced AKI is complex and still poorly understood. While it certainly involves factors like inflammation, oxidative stress, microvascular dysfunction, and tubular epithelial cell insult [6, 7], disseminated intravascular coagulation (DIC) might also contribute to AKI development. DIC results from an excessive activation of coagulation, along with a defect in anticoagulant and fibrinolytic regulatory systems [8, 9], and thus leads to disseminated microthrombosis, which might contribute to organ dysfunction during septic shock [10, 11]. As 30–40% of patients with septic shock develop DIC, which is associated with severity and increased mortality rate [12, 13], it might be a considerable contributor to AKI development.

However, few studies investigated the association between DIC and AKI. A higher rate of AKI has been suggested in septic shock patients with DIC, but this possible association was not analyzed with adjustment of confounding variables, mainly patient severity [14, 15].

This study, therefore aimed at investigating the association between DIC and AKI among patients with septic shock with a special emphasis on AKI occurrence, severity, and evolution.

## Methods

### Design of the study

A prospective cohort study was conducted between July 2013 and March 2019, in an ICU of Strasbourg University Hospital (France).

Criteria for inclusions were patients aged 18 years and older, admitted to the ICU for septic shock according to the Sepsis-2 definition were screened. Patients had to be included in the 12 h following vasopressor initiation. Patients with moribund status at screening phase, or patients with limitation of life-sustaining therapies at admission have been excluded. Patients under legal protection (inability to provide consent, incarceration…) have been excluded. Patients already enrolled in the present study have been excluded in case of readmission, and patients who developed shock later during the ICU stay were not screened.

Furthermore, patients with a history of terminal chronic kidney disease (stage 5 of the chronic kidney disease (CKD) definition [16], under chronic renal replacement therapy or kidney transplant recipients) were excluded. To delineate the association between DIC and AKI and its severity, patients in whom DIC was diagnosed after AKI reached its higher stage were excluded.

### Data collection

The following data were prospectively recorded: age, sex, body mass index, significant medical history, and the Charlson index [17]. Acute condition was characterized by: severity with the SAPSII and SOFA scores [18, 19], hemodynamic parameters (mean arterial pressure, urine output), source of infection, nosocomial or community-acquired infections and available microbiological data were collected. We registered all the supporting therapies required during the ICU stay [mechanical ventilation, vasopressors, fluid therapy, renal replacement therapy (RRT)] and their duration. Mortality was assessed at the end of ICU and hospital stay, and at day 28.

### Definitions

- AKI occurrence and severity were defined according to the Kidney Disease Improving Global Outcome (KDIGO) classification [20]. Stage 1 (KDIGO1) was considered when serum creatinine increased by at least 26 µmol/L from baseline creatinine or 1.5 to 1.9 times from the baseline creatine, or if urine output was below 0.5 mL/kg/h for 6–12 h. Stage 2 (KDIGO2) was reached if serum creatinine increased to more than twofold from baseline or if urine output was less than 0.5 mL/kg/h for at least 12 h. Stage 3 (KDIGO 3) represented the most severe form of AKI and was defined as an increase in serum creatinine to more than threefold from baseline, or upper than 354 µmol/L, or need for RRT or urine output below 0.3 mL/kg/h for at least 24 h or anuria for at least 12 h.
- Baseline serum creatinine: for each patient, when available, pre-admission serum creatinine was considered within a time period of a minimum of 7 days and a maximum of 1 year from hospital admission. If unavailable, when serum creatinine was elevated at admission, baseline serum creatinine was extrapolated using the MDRD formula [21] assuming that baseline estimated Glomerular Filtration Rate (GFR) is 75 mL/min/1.73 m^2^, as suggested by guidelines [20]. Patients were considered with CKD when baseline GFR was below 60 mL/min/1.73 m^2^.
  To describe the evolution of AKI, we collected as far as possible criteria for acute kidney disease (AKD), that was defined as a persistent AKI (stage 1 or higher of the KDIGO classification) for more than 7 days. Furthermore, at the end of the hospital stay, criteria for the composite endpoint for Major Adverse Kidney Event (MAKE) at the end of hospitalization were collected for each patient. MAKE was defined as the composite criteria of death, need for RRT or worsened kidney function (a twofold increase in serum creatinine level from baseline) at the end of hospital stay [22].
- DIC diagnosis was based on the International Society on Thrombosis and Haemostasis (ISTH) score [23], and, accordingly, a score upper or equal to 5 was retained for the diagnosis of DIC. Biological tests for the diagnosis of DIC were performed at ICU admission and on a daily basis until day 7.
- Nephrotoxic drugs: all the potential nephrotoxic drugs administered before ICU admission and before AKI occurrence during the ICU stay, among a predefined list, based on a previous work [24] were identified in patient’s record (see Additional file 1).

### Ethical concerns

The design of the study was approved by the ethic committee of Strasbourg (*Comité de Protection des Personnes* N° 12/35, DC-2012-1633). Before inclusion, written informed consent was obtained from all patients. If patients were unable to provide informed consent, it was obtained from their next of kin or another surrogate decision-maker, as appropriate. Post hoc consent was obtained as soon as possible in these patients.

### Statistic

Quantitative data were expressed as mean, standard deviation, median, and interquartile range (IQR) for parametric and non-parametric distributions, respectively, and were compared using Student’s *t*-tests or Wilcoxon rank-sum tests as appropriate. Qualitative variables were compared using *χ*^2^ or Fisher’s exact test.

To identify factors associated with AKI occurrence, severity, and evolution (MAKE criteria at the end of hospital stay), as odds ratios, logistical regression models were performed. In the first step, univariate analyses were conducted for every baseline characteristic variable, independently of each other. In the second step, multivariate models were built using variables with clinical relevance and/or with *p*-value < 0.2 in univariate analysis. When some covariates were strongly correlated, the most associated was kept in the multivariate model. Some continuous variables (norepinephrine and fluid administration) were transformed into qualitative variables according to clinically relevant values.

As AKI occurrence and severity in the ICU depends on mortality and ICU length of stay, we performed a survival analysis in ICU free from stage 3 AKI occurrence in patients with and without DIC (DIC diagnosis was considered as baseline) during the first 7 days. Survival without stage 3 AKI was estimated by the Kaplan–Meier method and was compared between the two groups with the use of a log-rank test.

## Results

### Baseline characteristics

**Fig. 1:**
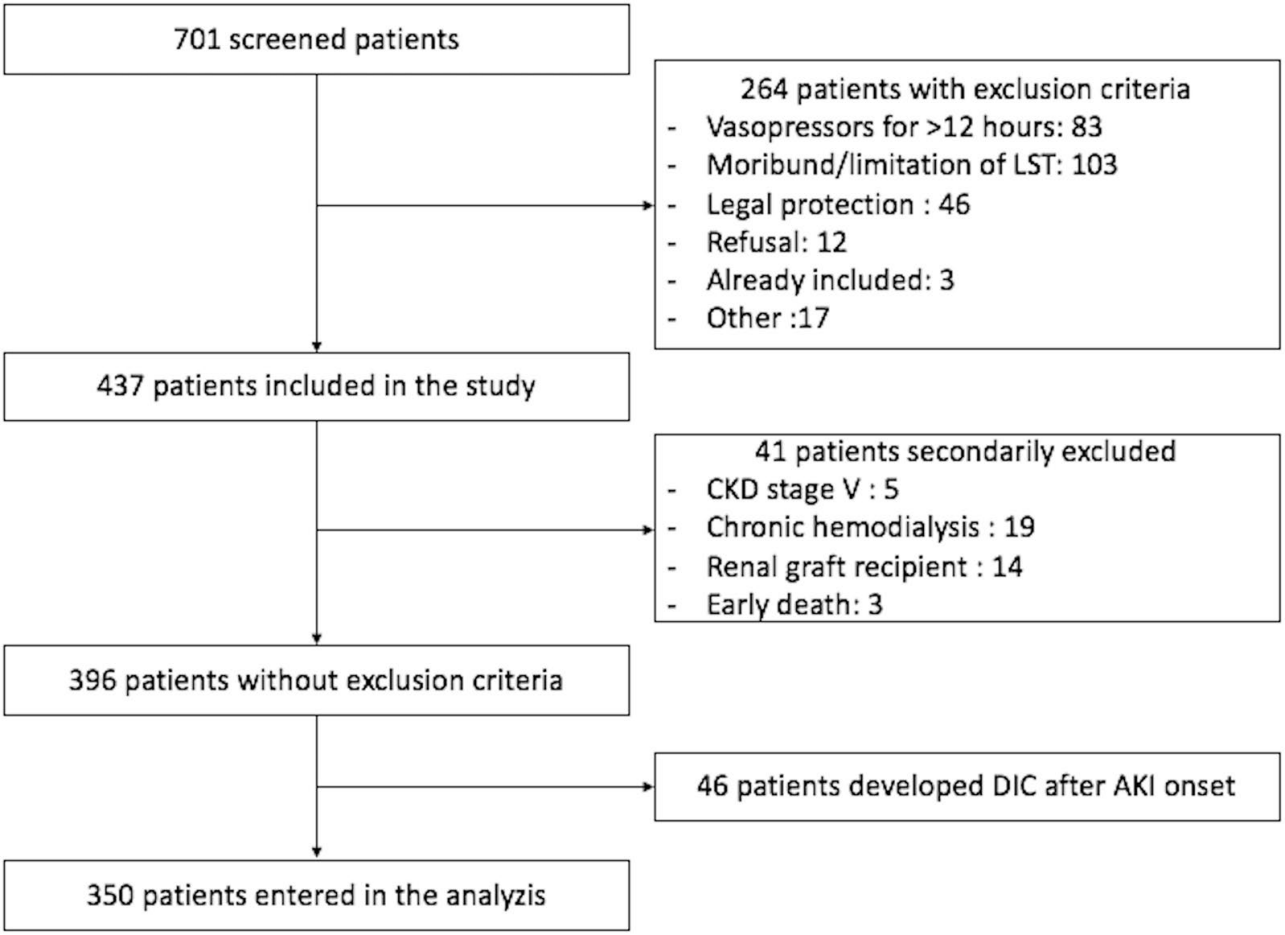
Flowchart of the study

From the 437 patients of the initial cohort, 350 patients were eligible and included in the study. Reasons for patient exclusion are presented in the flowchart (Fig. 1). Baseline characteristics are presented in Table 1.

**Table 1:** Baseline characteristics of the cohort, according to the occurrence of disseminate intravascular coagulation

Characteristics	Total (*n* = 350)	Patients with DIC (*n* = 129)	Patients without DIC (*n* = 221)	*p*
Age (years), mean ± sd	67 ± 14	66 ± 15	68 ± 13	0.115
Med [IQR]	69 [60–77]	68 [57–77]	70 [60–77]	0.179
Male sex, *n* (%)	227 (64.9)	76 (58.9)	151 (68.3)	0.075
SAPS II (points), mean ± sd	61 ± 19	64 ± 20	59 ± 18	***0.011***
Med [IQR]	58 [48–73]	64 [49–78]	56 [47–69]	***0.010***
SOFA (points), mean ± sd	11 ± 3	13 ± 3	10 ± 3	** < *0.001***
Med [IQR]	11 [9–13]	13 [10–15]	9 [8–12]	** < *0.001***
Preexisting conditions, *n* (%)				
Chronic hypertension	206 (58.9)	67 (51.9)	139 (62.9)	***0.044***
Diabetes	103 (29.4)	28 (21.7)	75 (33.9)	***0.015***
Chronic heart failure	23 (6.6)	9 (7.0)	14 (6.3)	0.815
Ischemic heart disease	27 (7.7)	7 (5.4)	20 (9.0)	0.220
Chronic kidney disease	49 (14.0)	9 (7.0)	40 (18.1)	***0.004***
Liver cirrhosis	14 (4.0)	11 (8.5)	3 (1.4)	** < *0.001***
Chronic obstructive pulmonary disease	65 (18.6)	16 (12.4)	49 (22.2)	***0.023***
Cancer	65 (18.6)	18 (13.9)	47 (21.3)	0.090
Charlson index, mean ± sd	2.6 ± 3.0	2.7 ± 2.9	2.6 ± 2.9	0.847
Med [IQR]	1 [0–4]	2 [1–4]	2 [1–4]	0.227
Basal serum creatinine (µmol/L), mean ± sd	83.3 ± 35.8	77.6 ± 24.9	87.5 ± 40.5	***0.013***
Med [IQR]	76 [62–93]	75 [61–91]	78.2 [63–95]	0.137
Back calculated, *n* (%)	106 (30.3)	47 (36.4)	59 (26.7)	0.056
Infection, *n* (%)				
Source of infection				
Lung	123 (35.1)	41 (31.8)	82 (37.1)	0.314
Abdominal	53 (15.1)	25 (19.4)	28 (12.7)	0.091
Urinary tract	72 (20.6)	27 (20.9)	45 (20.4)	0.899
Bloodstream infection	90 (25.7)	38 (29.5)	52 (23.5)	0.221
Other/unknown	90 (25.7)	28 (21.7)	62 (28.1)	0.190
Nosocomial infection	31 (8.9)	9 (7.0)	22 (10.0)	0.344
Immunosuppression	39 (11.1)	13 (10.1)	26 (11.8)	0.628
Involved bacteria				
Cocci gram positive	122 (34.9)	40 (31.0)	82 (37.1)	0.248
Bacillus gram negative	156 (44.6)	64 (49.6)	92 (41.6)	0.147
Other/unknown	72 (20.6)	25 (19.4)	47 (21.3)	0.673
Organ-support at inclusion				
Norepinephrine, *n* (%)	346 (98.9)	126 (97.7)	220 (99.5)	0.112
Epinephrine, *n* (%)	36 (10.3)	18 (14.0)	18 (8.1)	0.084
Dobutamine, *n* (%)	66 (18.9)	26 (20.2)	40 (18.1)	0.635
Norepinephrine highest dose^a^ (µg/kg/min), mean ± sd	1.0 ± 1.0	1.3 ± 1.4	0.8 ± 0.8	** < *0.001***
Med [IQR]	0.6 [0.3–1.2]	0.9 [0.4–1.9]	0.5 [0.3–0.9]	** < *0.001***
Fluid therapy before inclusion (liters), mean ± sd	0.8 ± 1.0	0.9 ± 0.9	0.8 ± 1.0	0.330
Med [IQR]	0.5 [0–1.3]	0.8 [0–1.5]	0.4 [0–1.0]	0.102
Invasive mechanical ventilation, *n* (%)	306 (87.4)	120 (93.0)	186 (84.2)	***0.016***
Mean diuresis at day 1 (mL/kg/h), mean ± sd	0.7 ± 0.8	0.7 ± 0.8	0.8 ± 0.8	0.294
Med [IQR]	0.5 [0.2–1]	0.5 [0.2–0.9]	0.5 [0.2–1.1]	0.283

Bolditalic characters were proposed to illustrate values who reached statistical significancy, i.e. *p* < 0.05

Data are expressed as mean ± standard deviation (sd), or median (Med) with interquartile range (IQR) for quantitative variables and as number and percentages for qualitative variables [*n* (%)]. The Chi-square test was used for qualitative data. Quantitative data were compared by *t*-test for mean comparison and Mann–Whitney test for median comparison

DIC, disseminated intravascular coagulation; SAPS II, Simplified Acute Physiologic Score II; SOFA, Sequential Organ Failure Assessment

^a^Norepinephrine highest dose was collected during the first 24 h after the beginning of infusion

Included patients were predominantly males, a median age of 69 years old. Patients were seriously ill with a median SAPS II of 58 (48–73) points. Cardiovascular history was the most frequent comorbidity. Baseline serum creatinine was unavailable in 106 patients (30.3%) and was extrapolated through MDRD equation calculation as planned by the study design. Infections responsible for septic shock were mainly community-acquired ones. All patients required vasopressor treatment, with norepinephrine as the first-used agent, and most of them (*n* = 306, 87.4%) required mechanical ventilation.

Among the 350 patients included, 129 patients developed DIC, while 221 did not. DIC occurred at day 1 in 92 patients, at day 2 for 22 patients, at day 3 for 7 and between day 4 and 7 for 8 patients. Coagulation tests at admission and at DIC diagnosis are presented in Additional file 2: Table S1. No other DIC provider than sepsis was present in patients with DIC.

### Patients with DIC were more seriously ill

As shown in Table 1, although patients with DIC were less comorbid, their severity scores (SOFA and SAPSII) at ICU admission were higher. There were no differences in the source of infection, communautary or nosocomial nature of infection and microbiological findings between patients with or without DIC. As a consequence of higher severity, patients with DIC required higher doses of norepinephrine and more frequently invasive mechanical ventilation.

Among the 129 patients with DIC, 11 suffered from cirrhosis, of whom 6 met the ISTH criteria for DIC without the prothrombin time component. Among the remaining five patients, a decrease of at least 30% in platelet count over time was taken into consideration as an additional criteria [25]. Similarly, for patients treated with oral anticoagulation with vitamin K antagonists, DIC diagnosis was retained only if they met the ISTH criteria without the prothrombin time component and a positive JAAM 2016 score.

### Patients with DIC had higher mortality- and AKI-rates

Patients with DIC had a higher mortality rate when compared to patients without DIC (43.3% vs. 26.2%, respectively, *p* < 0.01, for in-ICU mortality).

**Fig. 2:**
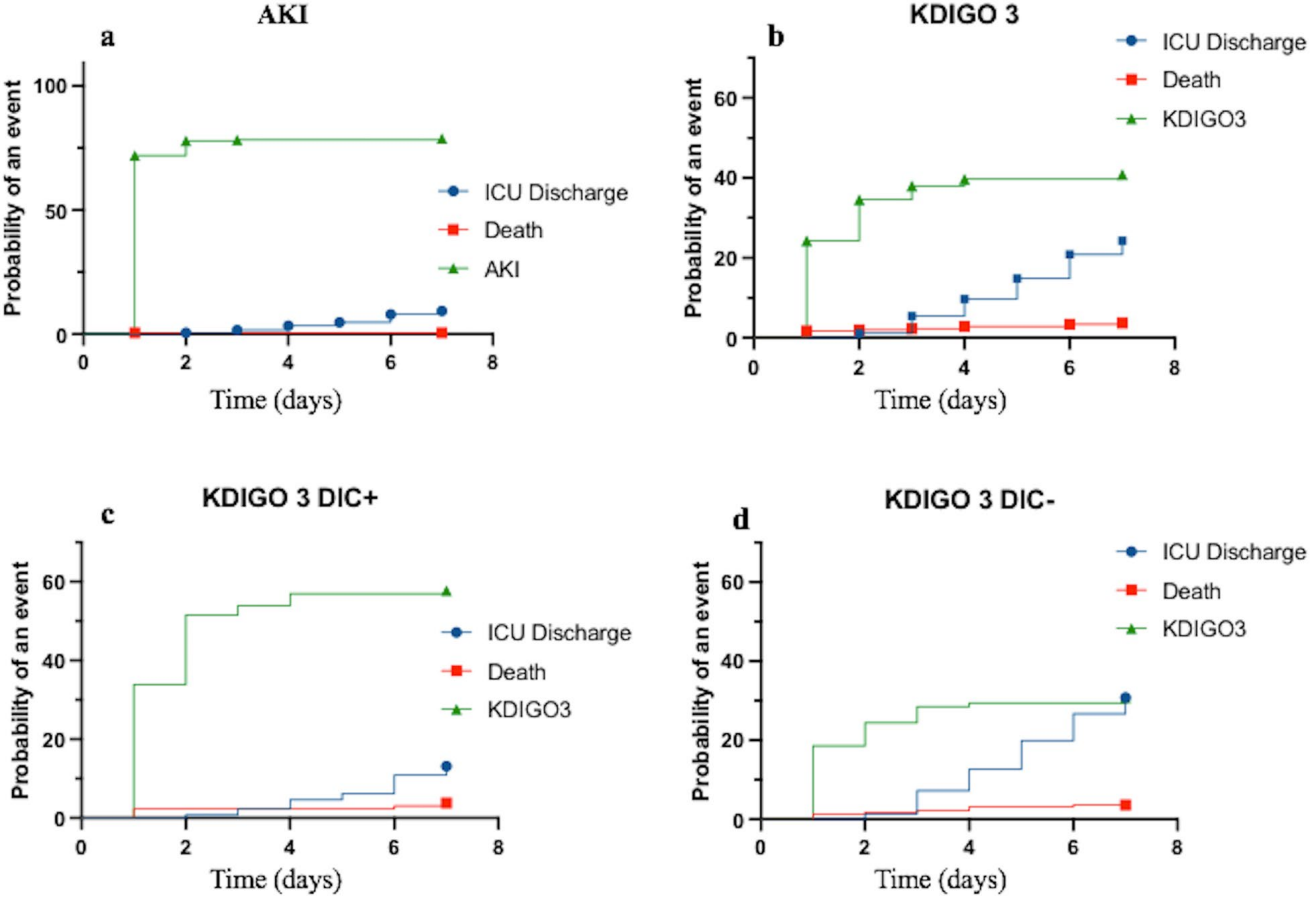
Cumulative incidence functions of competing events: acute kidney injury in the ICU (**a**), AKI KDIGO3 in the ICU (**b**), ICU death and ICU discharge. **b** AKI KDIGO unstratified and according to DIC status (DIC [KDIGO3 DIC +] **c** and no DIC [KDIGO3 DIC −] **d**). AKI, acute kidney injury; DIC, disseminated intravascular coagulation; KDIGO, Kidney Disease Improving Global Outcome (KDIGO 3: threefold increase from creatinine baseline or creatinine > 354 µmol/L; or Renal replacement Therapy or urine output < 0.3 mL/kg/h during 24 h or anuria during more than 12 h)

AKI occurred in 276 patients (78.9%). In 256 patients, AKI began within the first 24 h following ICU admission, and reached the higher stage at day 1 for 185 patients, at day 2 for 62 patients, at day 3 for 13 patients and between day 4 and 7 for the remaining 16 patients. AKI was more frequent in patients with DIC: 112 (86.8%) vs. 164 (74.2%) in patients without DIC (*p* = 0.005) (see Fig. 2 and Additional file 3: Fig. S1a). AKI and DIC occurred at the same day in 95 of the 112 patients.

### Patients with DIC were more likely to develop severe AKI and fulfill MAKE criteria

Patients with DIC were more likely to develop severe AKI, reaching stage 3 of the KDIGO classification in 73/129 patients with DIC (58.1%) *versus* 68/221 patients without DIC (30.8%), respectively (*p* < 0.001) (see Fig. 2 and Additional file 3: Fig. S1b). AKI stage 3 and DIC occurred at the same day in 57 of the 73 patients.

Among patients with stage 3 AKI, 108 required RRT during their ICU stay (61/129 patients with DIC (47.3%) vs. 47/221 patients without DIC (21.3%), *p* < 0.001).

**Fig. 3:**
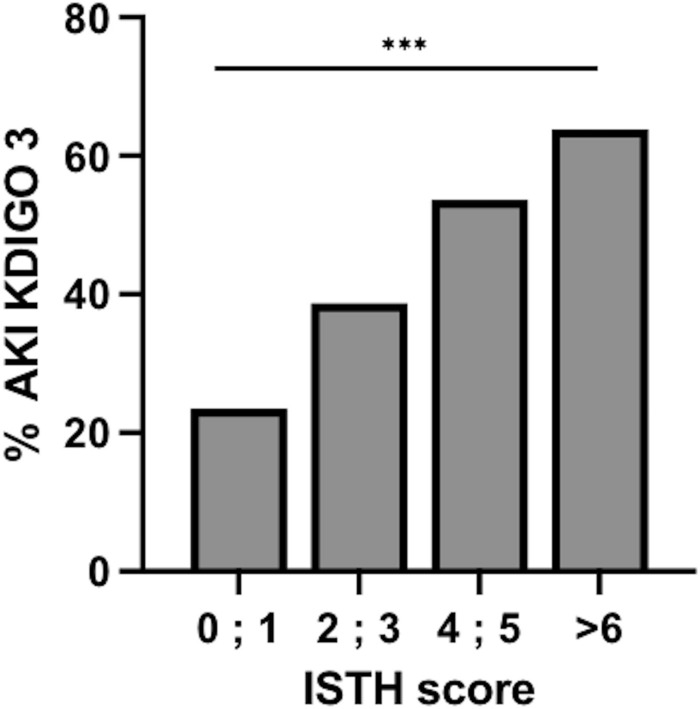
Proportion of patients with acute kidney injury stage 3 of the KDIGO classification according to the value of the ISTH score. AKI, acute kidney injury; ISTH, International Society on Thrombosis and Haemostasis; KDIGO, Kidney Disease Improving Global Outcome

We found that the higher the ISTH score, the higher the proportion of patients with stage 3 AKI (*p* < 0.001) (Fig. 3). The association between ISTH score and other outcomes is presented in Additional file 2: Table S2.

**Fig. 4:**
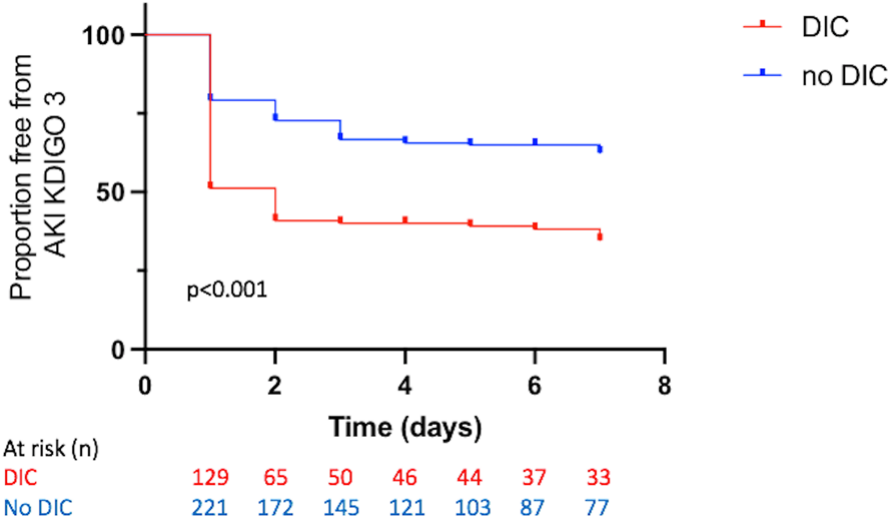
Probability of survival without acute kidney injury stage 3 of the KDIGO classification. AKI, acute kidney injury; DIC, disseminated intravascular coagulation; KDIGO, Kidney Disease Improving Global Outcome. Kaplan–Meier curves of the probability of survival without AKI reaching the stage 3 of the KDIGO classification during the first 7 days, according to the presence of DIC or not. *p* < 0.001 between groups (log-rank test)

The Kaplan–Meier curve analysis assessing the likelihood of developing stage 3 AKI of the KDIGO classification showed a higher risk for patients with DIC as compared to those without DIC, displaying a hazard ratio of 2.05 (1.48–2.85), *p* < 0.001 (log-rank test) (Fig. 4) (data were censored for deaths and leaving alive from ICU).

AKD was assessed in 236 patients, as those who died before day 7 after admission were not eligible for this evaluation. Among patients with available renal function evaluation after day 7, 74 experienced AKD (31.4%), of whom 34 had DIC, and 40 had not DIC [34/75 (45.3%) vs. 40/161 (24.8%), *p* = 0.002 *χ*^2^ test]. At the end of hospital stay, 71 patients (55%) with DIC fulfilled the MAKE criteria as compared to 78 patients (35.3%) without DIC (*p* < 0.001).

### After adjustment for cofounders, DIC remained significantly associated with AKI severity and need for renal replacement therapy

After adjustment for cofounders, DIC was still significantly associated with a higher risk of KDIGO 3 AKI, with an odds ratio (OR) of 2.74 [IC 95% (1.53–4.91), *p* < 0.001] (Table 2). Other factors significantly associated with KDIGO3 AKI were a history of CKD [OR 2.42 (1.03–5.68), *p* = 0.043], higher SAPS II [OR 1.06 (1.04–1.07), per 1 point increment *p* < 0.001] and age [OR 0.97 (0.95–0.99), *p* = 0.011]. An association between DIC and RRT requirement was also found [OR 2.83 (1.53–5.20), *p* < 0.001] (Table 3). When considering AKI—whatever its stage—DIC occurrence was no longer associated with AKI occurrence after multivariate analysis, nor with MAKE at the end of the hospital stay (Additional file 2: Tables S3 and 4).

**Table 2:** Univariate and multivariate analysis of factors associated with acute kidney injury meeting the criteria for the stage 3 of the KDIGO classification

	Univariate analysis			Multivariate analysis		
	OR	IC 95%	*p*	OR	IC 95%	*p*
DIC	3.12	1.99–4.91	** < *0.001***	2.74	1.53–4.91	** < *0.001***
Age (per 1-year increment)	1.0	0.99–1.02	0.645	0.97	0.95–0.99	***0.011***
Male sex	0.96	0.62–1.50	0.865	–	–	–
SAPS II (per 1pt increment)	1.06	1.05–1.08	** < *0.001***	1.06	1.04–1.07	** < *0.001***
SOFA (per 1pt increment)	1.44	1.31–1.57	** < *0.001***	–	–	–
Chronic hypertension	1.80	1.16–2.81	***0.009***	1.75	0.95–3.20	0.071
Diabetes	1.56	0.98–2.48	0.059	1.41	0.77–2.60	0.265
Chronic heart failure	1.35	0.58–3.16	0.483	–	–	–
CKD	1.78	0.97–3.26	0.063	2.42	1.03–5.68	***0.043***
Liver cirrhosis	2.71	0.89–8.27	0.079	1.00	0.26–3.91	0.996
Cancer	0.68	0.38–1.22	0.194	0.52	0.25–1.11	0.091
COPD	0.82	0.47–1.42	0.475	–	–	–
Source of infection						
Lung	1.15	0.74–1.80	0.532	–	–	–
Abdominal	1.77	0.99–3.19	0.056	1.66	0.80–3.43	0.175
Urinary tract	0.90	0.53–1.53	0.703	–	–	–
Bloodstream infection	0.79	0.48–1.29	0.349	–	–	–
Nosocomial infection	0.78	0.36–1.68	0.525	–	–	–
Immunosuppression	1.13	0.58–2.22	0.713	–	–	–
Cocci gram positive	0.78	0.49–1.22	0.269	–	–	-
Bacillus gram negative	1.49	0.97–2.28	0.071	1.47	0.86–2. 45	0.161
Organ-support at inclusion						
Epinephrine	6.06	2.67–13.73	** < *0.001***	1.99	0.71–5.55	0.187
Dobutamine	1.71	1.00–2.92	0.052	1.28	0.63–2.59	0.494
Norepinephrine > 1 µg/kg/min at day 1	3.18	1.99–5.07	** < *0.001***	1.31	0.72–2.39	0.372
Fluid before inclusion > 1L	5.57	1.21–17.18	***0.025***	4.34	0.82–23.1	0.085
Invasive mechanical ventilation	5.13	2.11–12.50	** < *0.001***	2.50	0.86–7.27	0.092
Nephrotoxic drugs, yes	0.52	0.19–1.43	0.207	0.77	0.18–3.23	0.724
Nephrotoxic drugs	1.06	0.89–1.25	0.514	–	–	–

Bolditalic characters were proposed to illustrate values who reached statistical significancy, i.e. *p* < 0.05

CKD, chronic kidney disease; COPD, chronic obstructive pulmonary disease; DIC, disseminated intravascular coagulation; OR, odds ratio; SAPS II, Simplified Acute Physiologic Score II; SOFA, Sequential Organ Failure Assessment

**Table 3:** Univariate and multivariate analysis of factors associated with renal replacement therapy requirement during the ICU stay

	Univariate analysis			Multivariate analysis		
	OR	IC 95%	*p*	OR	IC 95%	*p*
DIC	3.32	2.07–5.33	** < *0.001***	2.82	1.53–5.20	***0.001***
Male sex	0.89	0.55–1.42	0.62	–	–	–
Age, (per 1-year increment)	1.01	0.99–1.02	0.364	0.97	0.95–1	***0.048***
SAPS II (per 1pt increment)	1.06	1.04–1.08	** < *0.001***	1.06	1.04–1.07	** < *0.001***
SOFA (per 1pt increment)	1.44	1.31–1.58	** < *0.001***	–	–	–
Chronic hypertension	2.05	1.26–3.32	***0.004***	2.16	1.11–4.20	***0.023***
Diabetes	1.57	0.97–2.56	0.068	1.36	0.73–2.54	0.337
Chronic heart failure	1.21	0.50–2.95	0.674	–	–	–
CKD	1.67	0.90–3.11	0.106	1.70	0.74–3.90	0.208
Liver cirrhosis	4.31	1.41–13.18	0.01	2.01	0.50–7.89	0.325
Cancer	0.64	0.33–1.21	0.168	0.55	0.25–1.24	0.15
COPD	0.99	0.56–1.78	0.986	–	–	–
Source of infection						
Lung	0.84	0.52–1.36	0.474	–	–	–
Abdominal	1.74	0.95–3.18	0.071	1.58	0.76–3.31	0.221
Urinary tract	0.83	0.47–1.48	0.526	–	–	–
Bloodstream infection	0.71	0.41–1.21	0.208	–	–	–
Nosocomial infection	0.76	0.33–1.76	0.525	–	–	–
Immunosuppression	1.14	0.56–2.31	0.723	–	–	–
Cocci gram positive	0.59	0.36–0.97	0.037	0.53	0.29–0.97	***0.04***
Bacillus gram negative	1.37	0.87–2.16	0.173	1.15	0.62–1.96	0.747
Organ-support at inclusion						
Epinephrine	4.77	2.31–9.83	** < *0.001***	1.32	0.52–3.36	0.556
Dobutamine	1.6	0.92–2.79	0.097	1.12	0.55–2.31	0.75
Norepinephrine > 1 µg/kg/min at day 1	3.75	2.31–6.08	** < *0.001***	0.57	0.31–1.07	0.079
Fluid before inclusion > 1L	2.31	0.73–7.35	0.155	0.67	0.37–1.22	0.193
Nephrotoxic drugs, yes	0.73	0.26–2.07	0.557	–	–	–
Nephrotoxic drugs	1.09	0.91–1.33	0.336	1.02	0.80–1.30	0.871

Bolditalic characters were proposed to illustrate values who reached statistical significancy, i.e. *p* < 0.05

CKD, chronic kidney disease; COPD, chronic obstructive pulmonary disease; DIC, disseminated intravascular coagulation; OR, hazard ratio; SAPS II, Simplified Acute Physiologic Score II; SOFA, Sequential Organ Failure Assessment

## Discussion

In this prospective cohort of septic shock patients, DIC was associated with a near threefold increased risk of developing the more severe stage of AKI (KDIGO3) and of requiring RRT, which highly suggest that DIC worsens the prognosis of septic shock patients and contributes to multiple organ failure and its severity. However, after adjustment, DIC was no longer associated with AKI or MAKE criteria.

These findings are of interest. Indeed, if DIC occurrence was previously reported to be an independent factor associated with both severity and mortality in patients with septic shock [11, 12, 26, 27], the link between AKI and DIC had been poorly investigated. In a retrospective cohort of patients with septic shock caused by intra-abdominal infection, Xu et al*.* [28] showed that some coagulation biomarkers (aPTT, prothrombin time, and D-dimers) on ICU admission were significantly associated with AKI after multivariable logistic regression analysis, suggesting that coagulation activation might play a role in AKI development. Another retrospective study suggested the link between DIC and AKI, as biomarkers of endothelial injury (such as soluble thrombomodulin, E-selectin, protein C, and plasminogen activator inhibitor-1) were associated with AKI occurrence [29]. In this study, DIC was associated with AKI in univariate analysis, but no adjustment was made with cofounders, and the timing of DIC and AKI is not described.

In acute and chronic renal disease, the coagulation system and coagulation protease-dependent signaling might be altered [30]. Coagulation regulators and receptors both play a pivotal role in hemostasis and non-hemostatic functions in the kidneys. It has indeed been shown that coagulation proteases are able to alter the function of renal cells via protease-activated receptors (PARs) and co-receptors, while activated protein C would have nephroprotective effects that are at least partly independent of its anticoagulant function. It is therefore not surprising that excessive coagulation activation might alter renal function.

In a monocentric retrospective study including 582 critically ill patients, overt-DIC was associated with AKI occurrence in univariate analysis, but not in multivariate analysis, and DIC was associated with higher mortality in multivariate analysis [31]. Other studies highlighted this association [14, 15, 32]; however, none of these studies provided clear information regarding: (1) the timing of AKI development and (2) AKI severity. The design of our study is therefore original regarding these two points. Indeed, we have tried to assess the early course of both AKI and DIC during septic shock. With this in mind, we have included patients who met AKI and DIC criteria at the same timeframe.

From a pathophysiological point of view, it is now admitted that AKI related to septic shock is a multifactorial disease, not only attributable to kidney hypoperfusion. AKI associated with sepsis involves multiple mechanisms, including oxidative stress, inflammation, tubular cell adaptation to injury, renal hemodynamic alterations, and microcirculation dysfunction [7, 33]. Kidney microcirculation dysfunction during sepsis is related to both an alteration in renal blood flow [34] and to endothelial injury [35, 36], particularly in peritubular capillaries [37]. As a result, endothelial biomarkers, such as soluble thrombomodulin for example, were reported to be independent predictive biomarkers for AKI [29], even if this must be tempered as soluble thrombomodulin is excreted by kidneys. Such interplay between AKI, and endothelial dysfunction reinforces the hypothesis that AKI and DIC might be related.

DIC indeed results from excessive activation of coagulation pathways associated with vascular endothelial damage, and hypofibrinolysis [10, 38], that ultimately results in disseminated microthrombi formation that impairs microcirculation. Thus, DIC represents a major contributor to the development or worsening of organ failures [10, 11]. However, histopathologic features do not support such association. Indeed, in *post-mortem* renal biopsies of 19 patients with septic shock, arteriolar thromboses were found only in 4 patients, without relationship with the presence of DIC or not [39].

The present study also suffers from some shortcomings: being monocentric, the generalization of the findings is unsure. The main limitation in the present study is that, despite the design of our study, DIC and AKI occurred at the same time in more than 80% of the population, and in 76% of the patients with AKI KDIGO3 and DIC.

Elsewhere, the high incidence of AKI (78.9%) limits the external validity of our study. This high incidence might, however, be explained by the severity of the included patients (median SAPS II 61 points), and by the definition of AKI cases, strictly according to the KDIGO definition, taking into account urine output criteria. Indeed, it has been reported in a large multicenter observational cohort that taken into account or not the diuresis component result in a large difference in the number of patients with sepsis-induced AKI diagnosis, with differences in general and renal evolution [40]. In addition, the epidemiology of sepsis-induced AKI is poorly reported [41]. However, in a post hoc analysis of the ProCESS trial, AKI occurred in 69% of septic shock patients during the first 7 days after admission in the ICU, closest to our findings, with lower severity (29.7% of patients with AKI KDIGO 3) [42]. This high incidence of AKI in patients with high severity scores might explain the absence of association between AKI and DIC after adjustment for cofounders, as a result of insufficient statistical power.

Unfortunately, renal evolution at day 7 was missing in 33% of the cohort, which precludes any conclusion with regard to renal evolution. To deal with this concern, we have collected criteria for MAKE classification, which was reported to be a relevant composite criterion to illustrate clinically meaningful adverse outcomes following AKI: new hemodialysis, death and persistent impaired renal function (with variable range for such definition).

The association between DIC and patients who fulfilled the MAKE criteria was not found after adjustment, but the time of this evaluation (end of hospitalization) is probably too early for a relevant evaluation of renal function evolution in patients who experienced septic shock.

Lastly, we have chosen the ISTH score [23] for DIC definition whereas other score such as the Japanese Association for Acute Medicine-DIC (JAAM-DIC) [43] were proposed to allow an earlier recognition of DIC in the setting of sepsis. If the optimal score for DIC diagnosis remain a matter of debate [44], today, the ISTH score is recommended by learned societies [45].

## Conclusion

In this prospective cohort of septic shock patients, DIC was strongly associated with the risk of KDIGO3 AKI, even after adjustment for severity and other relevant factors. Of course, this study cannot establish the causality of such relationship. If DIC indeed plays a role in the pathogenesis of AKI among patients with septic shock, forthcoming studies focusing on DIC should likely incorporate AKI as a significant outcome. Similarly, research endeavors investigating AKI within the context of sepsis and its risk factors should perhaps consider DIC as a potential contributing factor, a consideration that has not been addressed so far.

### Supplementary Information

**Additional file 1.** List of nephrotoxic drugs collected in patient's medical record.

**Additional file 2. Table S1**: Results of the coagulation test at inclusion and at diagnosis of disseminated intravascular coagulation. **Table S2**: Main outcomes of included patients, according to the value of the ISTH score. **Table S3**: Univariate and multivariate analysis of factors associated with Acute Kidney Injury occurrence during the ICU stay. **Table S4**: Univariate and multivariate analysis of factors associated with Major Adverse Kidney Events at the end of the hospital stay.

**Additional file 3. Fig. S1**: **a** Acute Kidney Injury occurrence according to the presence of disseminated intravascular coagulation. **b** Stages of Acute Kidney Injury (KDIGO classification), according to the presence of disseminated intravascular coagulation. AKI, acute kidney injury; DIC, disseminated intravascular coagulation; KDIGO, Kidney disease Improving global outcome (KDIGO 1: increase of 26.5 µmol/L from baseline creatinine or 1.5–1.9 fold increase from baseline or urine output < 0.5 mL/kg/h during 6–12 h; KDIGO 2, 2–2.9 fold increase creatinine from baseline or urine output < 0.5 mL/h during at least 12 h; KDIGO 3, 3 fold increase from creatinine baseline or creatinine > 354 µmol/L, or Renal replacement Therapy or urine output < 0.3 mL/kg/h during 24 h or anuria during more than 12 h).

## Data Availability

Datasets analyzed during the current study are available from the corresponding author on reasonable request.
