# Supplementary material for: Disseminated intravascular coagulation is strongly associated with severe acute kidney injury in patients with septic shock
Source: Ann Intensive Care. 2023 Dec 1;13:119. doi: 10.1186/s13613-023-01216-8 (PMC10692023; doi:10.1186/s13613-023-01216-8)
Supplement: Supplementary file 1 — Additional file 1. List of nephrotoxic drugs collected in patient's medical record. [file 13613_2023_1216_MOESM1_ESM.docx]

**Supplemental material.** Lists of nephrotoxic drugs collected in patient’s medical record.

***- Diuretics:***

Loop diuretics

Thiazide

Aldosterone antagonists

***- Renin-angiotensin-aldosterone system inhibitors :***

Angiotensin converting enzyme inhibitors

Angiotensin II receptor blockers

***- Non Steroidal Anti Inflammatory***

***- Anti infectious therapies:***

Vancomycin

Aminosids

High Dose beta-lactamins

Rifampicin

Cotrimoxazole

Aciclovir

Adefovir, Cidofovir, Tenofovir, Indinavir

Amphotericin B

Foscavir

Ciclosporin, Tacrolimus

Methotrexate

Cisplatin

Lithium

***- Iodinated contrast medium***
