# Supplementary material for: Disseminated intravascular coagulation is strongly associated with severe acute kidney injury in patients with septic shock
Source: Ann Intensive Care. 2023 Dec 1;13:119. doi: 10.1186/s13613-023-01216-8 (PMC10692023; doi:10.1186/s13613-023-01216-8)
Supplement: Supplementary file 2 — Additional file 2. Table S1: Results of the coagulation test at inclusion and at diagnosis of disseminated intravascular coagulation. Table S2: Main outcomes of included patients, according to the value of the ISTH score. Table S3: Univariate and multivariate analysis of factors associated with Acute Kidney Injury occurrence during the ICU stay. Table S4: Univariate and multivariate analysis of factors associated with Major Adverse Kidney Events at the end of the hospital stay. [file 13613_2023_1216_MOESM2_ESM.docx]

**Supplemental Table 1**. Results of the coagulation test at inclusion and at diagnosis of disseminated intravascular coagulation.

| Characteristics | Patients with DIC  (n=129) | Patients without DIC  (n=221) | p |
| --- | --- | --- | --- |
| Platelet count (G/L), mean±sd  Med [IQR] | 135±107  116 [47-190] | 203±170  171 [113-254] | ***<0.01***  ***<0.01*** |
| Prothrombine Time, mean±sd  Med [IQR] | 54.9±24.4  56 [36-70] | 52±23.1  53.5 [34-67] | ***0.01***  ***0.02*** |
| Fibrinogen (g/L), mean±sd  Med [IQR] | 4.8±2.0  4.8 [3.5-6.2] | 5.5±2.1  5.5 [4.1-6.9] | ***<0.01***  ***<0.01*** |
| D-Dimer (µg/mL), mean±sd  Med [IQR] | 9.1±6.5  7.1 [3.6-14.4] | 5.4±5.4  3.3 [1.9-6.8] | ***<0.01***  ***<0.01*** |

*Data are expressed as mean ± standard deviation (sd), and median (Med) with interquartile range (IQR). Quantitative data were compared by t-test for mean comparison and Mann-Whitney test for median comparison.*

**Supplementary Table 2.** Main outcomes of included patients, according to the value of the ISTH score.

| Characteristics | ISTH ≤2  (n=115) | ISTH 3;4  (n=106) | ISTH 5;6  (n=82) | ISTH>6  (n=47) | p |
| --- | --- | --- | --- | --- | --- |
| Age (years), mean ± sd  Med [IQR] | 68.1 ± 12.9  69 [60-77] | 68.3 ± 12.6  70 [61-77] | 67.3±15.2  69 [59-79] | 62.9±14.9  61 [55-74] | 0.15 |
| SAPS II (points), mean ±sd  Med [IQR] | 56.5 ± 17.3  55 [47-64] | 61.4 ± 19  59 [47-73] | 63.7 ± 19.1  64 [49-78] | 65.5 ± 21.5  66 [49-81] | ***0.01*** |
| SOFA (points), mean ±sd  Med [IQR] | 9.8 ± 2.7  9 [8-11.5] | 10 ± 3.1  10 [8-12] | 12.5± 3.0  12 [10-14] | 13.1± 3.3  13 [11-16] | ***<0.01*** |
| **Organ-support at inclusion** | | | | | |
| Norepinephrine, n (%) | 114 (99.1) | 106 (100) | 80 (97.6) | 46 (97.9) | 0.40 |
| Epinephrine, n (%) | 8 (6.9) | 10 (9.4) | 9 (11.0) | 9 (19.1) | 0.14 |
| Dobutamine, n (%) | 23 (20) | 17 (16) | 18 (21.9) | 8 (17) | 0.46 |
| Norepinephrine highest dose* (µg/kg/min), mean ±sd  Med [IQR] | 0.7 ± 0.8  0.5 [0.3-0.8] | 0.8 ± 0.7  0.5 [0.3-1.1] | 1.3 ± 1.5  0.9 [0.4-1.5] | 1.2 ± 1.2  0.7 [0.3-2.1] | ***<0.01*** |
| Invasive Mechanical Ventilation, n (%) | 93 (80.9) | 93 (87.7) | 79 (96.3) | 41 (87.2) | 0.01 |
| **Outcomes** | | | | | |
| AKI, n (%) | 87 (75.6) | 77 (72.6) | 68 (82.9) | 44 (93.6) | ***0.02*** |
| RRT requirement, n (%) | 19 (16.5) | 28 (26.4) | 36 (43.9) | 25 (53.2) | ***<0.01*** |
| MAKE, n (%) | 35 (30.4) | 43 (40.1) | 41 (50) | 30 (63.8) | ***<0.01*** |
| In-ICU mortality, n (%) | 26 (22.6) | 32 (30.2) | 30 (36.6) | 26 (55.3) | ***<0.01*** |
| Death at day 28, n (%) | 27 (23.5) | 33 (31.1) | 37 (45.1) | 26 (55.3) | ***<0.01*** |

*AKI: acute kidney injury, ISTH: International Society on Thrombosis and Haemostasis,*

*Data are expressed as mean ± standard deviation (sd), or median (Med) with interquartile range (IQR) for quantitative variables and as number and percentages for qualitative variables [n (%)]. The Chi-square test was used for qualitative data and the Kruskal-Wallis test for quantitative data.*

**Supplemental Table 3**. Univariate and multivariate analysis of factors associated with Acute Kidney Injury occurrence during the ICU stay

|  | Univariate analysis | | | Multivariate analysis | | |
| --- | --- | --- | --- | --- | --- | --- |
|  | OR | IC 95% | p | OR | IC 95% | p |
| DIC | 2.29 | 1.29 - 4.25 | ***0.006*** | 1.91 | 0.95 - 3.79 | 0.069 |
| Male sex | 0.79 | 0.45 - 1.36 | 0.41 | - | - | - |
| Age, per 1year increment | 1.03 | 1.01 - 1.05 | ***0.001*** | 1.01 | 0.99-1.04 | 0.254 |
| SAPS II, per 1 point increment | 1.04 | 1.02 - 1.06 | ***<0.001*** | 1.02 | 1 - 1.04 | ***0.035*** |
| SOFA, per 1 point increment | 1.34 | 1.21 - 1.48 | ***<0.001*** | - | - | - |
| Chronic hypertension | 2.58 | 1.53 - 4.40 | ***<0.001*** | 1.69 | 0.88 - 3.24 | 0.114 |
| Diabetes | 2.27 | 1.22 - 4.51 | ***0.013*** | 1.61 | 0.77 - 3.39 | 0.208 |
| Chronic heart failure | 2.96 | 0.84 -18.8 | ***0.148*** | 2.42 | 0.46 - 12.5 | 0.292 |
| CKD | 3.41 | 1.32 -11.6 | ***0.023*** | 2.11 | 0.39 - 11.3 | 0.385 |
| Liver cirrhosis | 15.7 | 0.01 - 49051 | ***<0.001*** | 14.3 | 0.07 - 44637 | ***<0.001*** |
| Cancer | 0.69 | 0.37-1.33 | 0.252 | - | - | - |
| COPD | 0.58 | 0.32 -1.09 | ***0.079*** | 0.61 | 0.33 - 1.12 | 0.183 |
| **Source of infection** | | | | | | |
| Lung | 0.74 | 0.44 - 1.27 | 0.274 | - | - | - |
| Abdominal | 1.37 | 0.66 - 3.13 | 0.422 | - | - | - |
| Urinary tract | 1.14 | 0.61 - 2.25 | 0.692 | - | - | - |
| Bloodstream infection | 0.71 | 0.41 - 1.26 | 0.236 | - | - | - |
| Nosocomial infection | 1.13 | 0.47 - 3.14 | 0.799 | - | - | - |
| Immunosuppression | 1.04 | 0.48 - 2.53 | 0.919 | - | - | - |
| Cocci gram positive | 0.68 | 0.40 - 1.16 | ***0.154*** | 0.61 | 0.33 - 1.12 | 0.11 |
| Bacillus gram negative | 1.00 | 0.60 - 1.68 | 0.996 | - | - | - |
| **Organ-support at inclusion** | | | | | | |
| Epinephrine | 5.06 | 1.49 - 31.6 | ***0.028*** | 2.23 | 0.44 - 11.22 | 0.329 |
| Dobutamine | 1.63 | 0.82 - 3.55 | ***0.189*** | 1.12 | 0.47 - 2.64 | 0.802 |
| Norepinephrine <1µg/kg/min at day 1 | 0.27 | 0.13 - 0.56 | ***<0.001*** | 0.48 | 0.21 - 1.07 | 0.073 |
| Fluid before inclusion <1L | 0.41 | 0.24 - 0.70 | ***0.001*** | 0.45 | 0.25 - 0.826 | ***0.01*** |
| Invasive Mechanical Ventilation | 1.29 | 0.59 - 2.61 | 0.504 | - | - | - |
| Nephrotoxic drugs, yes | 0.52 | 0.08 - 1.92 | 0.394 | - | - | - |
| Nephrotoxic drugs, per 1 increment | 1.17 | 0.95 - 1.44 | 0.136 | 1.1 | 0.86 - 1.40 | 0.444 |

*CKD: Chronic Kidney Disease, COPD: Chronic obstructive pulmonary disease, DIC: Disseminated Intravascular Coagulation, OR: Odd-Ratio, SAPS II: Simplified Acute Physiologic Score II, SOFA : Sequential Organ Failure Assessment.*

**Supplemental Table 4**. Univariate and multivariate analysis of factors associated with Major Adverse Kidney Events at the end of the hospital stay.

|  | Univariate analysis | | | Multivariate analysis | | |
| --- | --- | --- | --- | --- | --- | --- |
|  | OR | IC 95% | p | OR | IC 95% | p |
| DIC | 2.24 | 1.44 - 3.50 | ***<0.001*** | 1.17 | 0.65 - 2.11 | 0.714 |
| Male sex | 0.97 | 0.62 - 1.51 | 0.885 | - | - | - |
| Age, per 1 year increment | 1.01 | 0.99 - 1.03 | 0.076 | 1 | 0.98 - 1.03 | ***0.714*** |
| SAPS II, per 1 point increment | 1.06 | 1.04 - 1.07 | ***<0.001*** | 1.04 | 1.02 - 1.06 | ***<0.001*** |
| SOFA, per 1 point increment | 1.22 | 1.13 - 1.31 | ***<0.001*** | - | - | - |
| Chronic hypertension | 1.29 | 0.84 - 1.99 | 0.244 | - | - | - |
| Diabetes | 0.72 | 0.45 - 1.15 | 0.166 | 0.43 | 0.23 - 0.81 | ***0.009*** |
| Chronic heart failure | 1.04 | 0.44 - 2.44 | 0.927 | - | - | - |
| CKD | 0.83 | 0.45 - 1.55 | 0.563 | 0.53 | 0.23 - 1.21 | 0.13 |
| Liver cirrhosis | 8.71 | 1.92 - 39.56 | ***0.005*** | 11.9 | 1.91 - 74.44 | ***0.008*** |
| Cancer | 1.32 | 0.76 - 2.31 | 0.322 | - | - | - |
| COPD | 0.69 | 0.40 - 1.21 | 0.196 | 0.81 | 0.41 - 1.59 | 0.542 |
| **Source of infection** | | | | | | |
| Lung | 0.98 | 0.63 - 1.53 | 0.935 | - | - | - |
| Abdominal | 1.04 | 0.58 - 1.88 | 0.895 | - | - | - |
| Urinary tract | 1.18 | 0.70 - 1.99 | 0.53 | - | - | - |
| Bloodstream infection | 1.5 | 0.93 - 2.43 | 0.099 | 1.93 | 1.07 - 3.50 | ***0.029*** |
| Nosocomial infection | 0.97 | 0.46 - 2.05 | 0.94 | - | - | - |
| Immunosuppression | 2.11 | 1.07 - 4.15 | ***0.031*** | 1.89 | 0.81 - 4.45 | 0.144 |
| Cocci gram positive | 1.23 | 0.79 - 1.92 | 0.357 | - | - | - |
| Bacillus gram negative | 1.24 | 0.81 - 1.90 | 0.319 | - | - | - |
| **Organ-support at inclusion** | | | | | | |
| Epinephrine | 8.19 | 3.31 - 20.27 | ***<0.001*** | 3.16 | 1.08 - 9.18 | ***0.035*** |
| Dobutamine | 1.96 | 1.14 - 3.37 | ***0.015*** | 1.12 | 0.60 - 2.07 | 0.726 |
| Norepinephrine <1µg/kg/min at day 1 | 0.394 | 0.25 - 0.63 | ***<0.001*** | 0.93 | 0.51 - 1.71 | 0.817 |
| Fluid before inclusion >1L | 1.04 | 0.68 - 1.6 | 0.85 | - | - | - |
| Nephrotoxic drugs, yes | 0.56 | 0.20 - 1.54 | 0.263 | - | - | - |
| Nephrotoxic drugs | 1.26 | 1.01 - 1.50 | ***0.008*** | 1.24 | 0.99 - 1.54 | 0.052 |
| AKI KDIGO 3: yes | 5.61 | 3.52 - 8.93 | ***<0.001*** | 3.85 | 2.11 - 7.02 | ***<0.001*** |

*AKI: Acute Kidney Injury, CKD: Chronic Kidney Disease, COPD: Chronic obstructive pulmonary disease, DIC: Disseminated Intravascular Coagulation, KDIGO: Kidney Disease Improving Global Outcomes, OR: Odd-Ratio, SAPS II: Simplified Acute Physiologic Score II, SOFA : Sequential Organ Failure Assessment.*
