# Supplementary figures and images for: Disseminated intravascular coagulation is strongly associated with severe acute kidney injury in patients with septic shock
Source: Ann Intensive Care. 2023 Dec 1;13:119. doi: 10.1186/s13613-023-01216-8 (PMC10692023; doi:10.1186/s13613-023-01216-8)

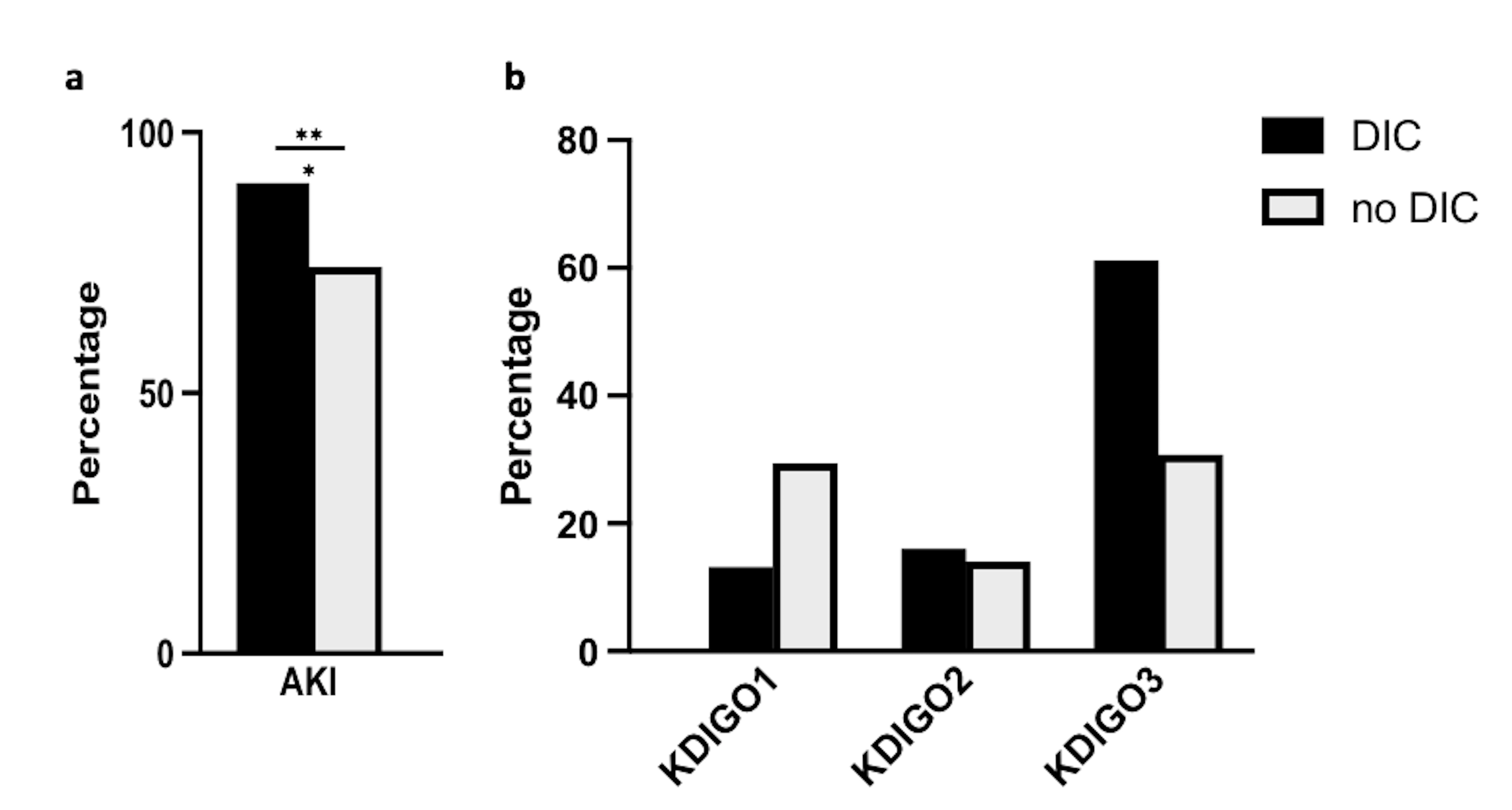

Supplement: Supplementary file 3 — Additional file 3. Fig. S1: a Acute Kidney Injury occurrence according to the presence of disseminated intravascular coagulation. b Stages of Acute Kidney Injury (KDIGO classification), according to the presence of disseminated intravascular coagulation. AKI, acute kidney injury; DIC, disseminated intravascular coagulation; KDIGO, Kidney disease Improving global outcome (KDIGO 1: increase of 26.5 µmol/L from baseline creatinine or 1.5–1.9 fold increase from baseline or urine output < 0.5 mL/kg/h during 6–12 h; KDIGO 2, 2–2.9 fold increase creatinine from baseline or urine output < 0.5 mL/h during at least 12 h; KDIGO 3, 3 fold increase from creatinine baseline or creatinine > 354 µmol/L, or Renal replacement Therapy or urine output < 0.3 mL/kg/h during 24 h or anuria during more than 12 h). [file 13613_2023_1216_MOESM3_ESM.png]
